# Influence of acute fasting on pain tolerance in healthy subjects: a randomised crossover study

**DOI:** 10.3389/fpain.2023.1153107

**Published:** 2023-09-11

**Authors:** Sophie A. Edwards, Sarah L. Martin, Timothy Rainey, Grace Whitaker, Darren C. Greenwood, Anthony Jones, Manoj Sivan

**Affiliations:** ^1^ The Human Pain Research Group, Division of Neuroscience and Experimental Psychology, University of Manchester, Manchester, United Kingdom; ^2^ Brain Health Imaging Centre, Centre for Addiction and Mental Health (CAMH), Toronto, ON, Canada; ^3^ Centro de Investigación y Desarrollo en Ingeniería en Salud, Universidad de Valparaiso, Valparaiso, Chile; ^4^ Leeds Institute for Data Analytics, University of Leeds, Leeds, United Kingdom; ^5^ Leeds Institute of Rheumatology and Musculoskeletal Medicine, University of Leeds, Leeds, United Kingdom

**Keywords:** chronic pain, eating, attention, mood, gender

## Abstract

**Background:**

Although chronic pain and obesity are global health crises with substantial healthcare costs, little is known about the relationship between pain perception and eating behaviours. Food consumption has been reported to provide an analgesic effect by the release of neurotransmitters modulating the pain network. However, whether short-term (acute) fasting affects pain perception remains unclear.

**Purpose:**

This study aimed to investigate the effect of acute fasting on pain perception and whether attention and mood changes drove the observed changes.

**Patients and methods:**

The cold pressor test (CPT) was used to investigate the pain tolerance of 25 healthy participants in both non-fasting and 12-h fasting sessions. They were randomised to either session with a crossover to the other after at least 24 h, with the experimenter blinded to the sessions. The pain tolerance was measured using a Stroop task in both attentive and distracted states. The Profile of Mood States (POMS) questionnaire was used to capture the mood, and a 10-point hunger scale was used to measure hunger. Mixed-effects models were used to investigate the influence of fasting and distraction on pain perception, accounting for the repeated measures.

**Results:**

Fasting reduced CPT pain tolerance, with fasting participants twice as likely to withdraw their hands early (hazard ratio = 2.4, 95% CI: 1.3–4.5). Though men tolerated CPT pain longer than women, there was no evidence that men responded to fasting differently than women (*p* = 0.9). In addition, no evidence supporting that fasting affected attention or mood was found. Nonetheless, it increased hunger scores by 2.7 points on a 10-point scale (95% CI: 1.2–4.2) and decreased blood glucose concentration levels by 0.51 mmol/L (95% CI: 0.19–0.84).

**Conclusion:**

Acute fasting reduces pain tolerance in the healthy participants, and this effect is independent of gender and attention or mood changes.

## Introduction

Chronic pain and obesity are two highly prevalent conditions that significantly contribute to the healthcare expenditure in most countries. Chronic pain (pain persisting for more than 3 months) affects more than one-third of the UK population (1). The risk of having chronic pain increases with body mass index (BMI) and doubles in obesity (2, 3). Pain is a complex process involving mechanisms in both the peripheral and central nervous systems. Several cognitive functions modulate pain processing in higher cortical regions, including attention, mood and emotion (4).

Food consumption has been reported to increase pleasure via modulating the levels of neurotransmitters, namely, dopamine (DA) and serotonin [5-hydroxytryptamine (5-HT)] (5). As these neurotransmitters are involved in the pain networks, increasing them by food intake could provide patients with a short-term improvement in their pain (6). This could help explain the association between obesity and chronic pain (3, 7). Studies have shown that food intake has an analgesic effect (8). However, the effect of short-term fasting on pain perception and tolerance in humans has not been well studied. In addition, whether acute fasting directly affects pain due to changes in neurotransmitters or alterations in cognitive functions remains unclear.

Fasting results in physiological changes, such as increased metabolism and heart rate changes and modulations in some psychological processes. Literature on the association between fasting and cognition is inconclusive (9). Although most studies concluded that fasting negatively impacts cognition and perception (10–12), some studies reported no change in cognition and attention (13, 14).

Animal studies have shown a biphasic response of fasting on pain with an initial increase in pain threshold (at 24 h) followed by a decrease in such threshold with prolonged fasting (15). So far, only one study has investigated the effects of food deprivation on pain perception in healthy humans (16), in which the researchers compared pain perception (induced by a pressure algometer) between an experimental group of women and a control group of non-fasted women after a 24-h fasting period. They found a significant reduction in pain threshold and tolerance accompanied by changes in the sympathovagal balance (decreased vagal activity). In addition, a negative mood state captured on a 19-item self-reported mood scale was significantly correlated with increased pain unpleasantness.

We aimed to investigate whether a shorter fasting period (12 h) can reduce pain tolerance. This was a more reasonable period of voluntary restriction of food intake experienced by individuals in real life. Through exploratory data analyses, we also aimed to explore whether changes in pain thresholds are mediated by gender or mood or attention changes.

## Materials and methods

### Ethical approval

The University of Manchester Research Ethics Committee granted ethical approval for this study. All participants provided informed consent before participation, in accordance with the World Medical Association Declaration of Helsinki (17).

### Participant recruitment

A total of 25 healthy individuals (13 men, 12 women) with a mean (SD) age of 21 (2) years were recruited from the University of Manchester. The inclusion criteria included an age range of 18–90 years, a BMI between 18 and 30 kg/m^2^ and English fluency. The exclusion criteria included a history of chronic pain, regular analgesic intake or analgesic intake 24 h before the study, medical conditions that might confound the cold pressor test (CPT) (such as Raynaud's phenomenon), neurological conditions (such as anxiety or depression), conditions that might affect performance in Stroop task (such as visual impairment or colour blindness), a history of an eating disorder, pregnancy/breastfeeding, being diabetic, and regular skipping of breakfast.

**Figure 1:**
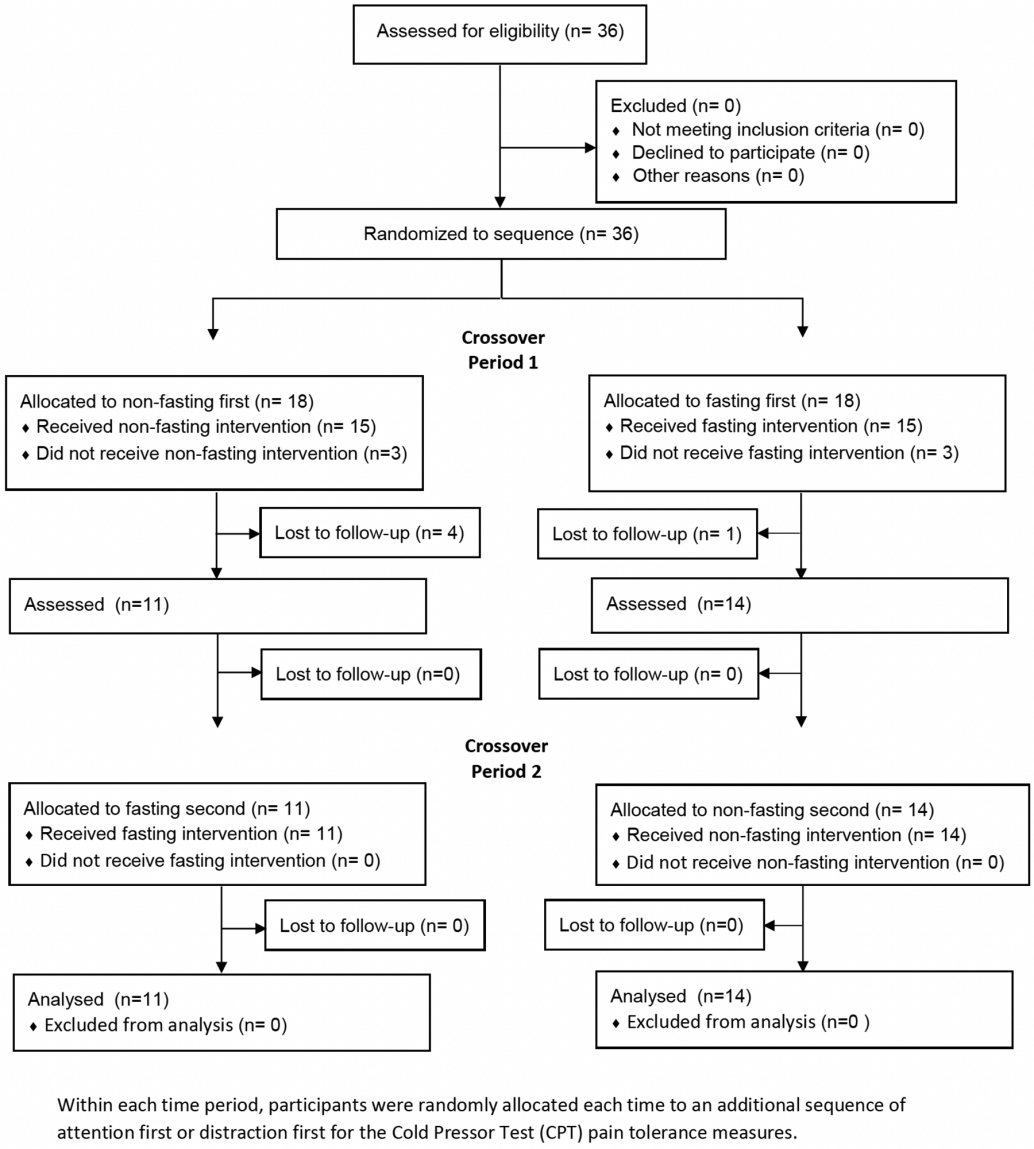
CONSORT flow diagram indicating the numbers of participants who were randomly assigned, received intended treatment and were analysed for the primary outcome by sequence and time period.

Figure 1 shows the CONSORT flow diagram indicating the numbers of randomly assigned participants, who received the intended intervention and were analysed for the primary outcome by sequence, with a crossover design between the fasted and non-fasted states.

### Fasting criteria and randomisation

The participants attended two study sessions, each with 24–168 h of washout periods, during which they consumed their usual diet. They were instructed to fast for 12 h before attending the first session and to eat normally before attending the second session. They were not allowed to consume food or alcohol for 12 h before each fasting session, except for caffeine to be consumed as usual. Simple randomisation was used to determine the order of fasting and non-fasting sessions. The researcher was blinded to the order, with participants receiving instructions via a code, so the experimenter was unaware whether the issue was fasted or not. It was not possible to blind the participants to the intervention. To limit variation between fasting durations, we scheduled all sessions between 09.00 h and 12.00 h.

### Experimental procedures

**Figure 2:**
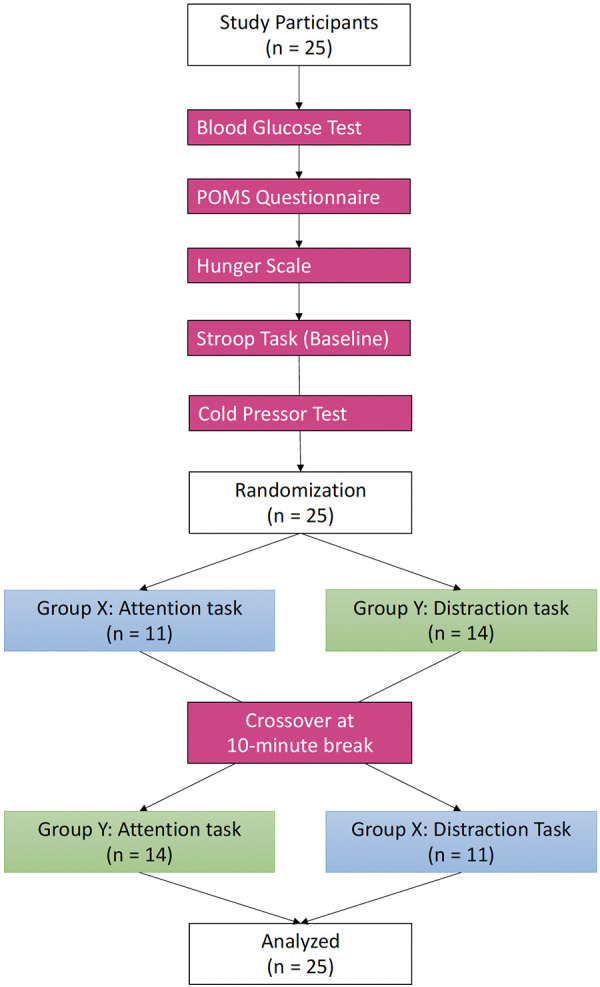
Experimental procedure diagram.

For each experimental visit, the participants completed the protocol (Figure 2), written in order of occurrence.

#### Blood glucose test

All participants had their capillary blood glucose levels and hunger scores measured during the fasted and non-fasted states. On arrival of the participants, a capillary blood glucose test was performed on their left index fingers using the Accu-Chek® Aviva Blood Glucose test kits (LOT495918, REF 06453970054; LOT 496694, REF 06453970018) and metre (Roche Diagnostics GmbH, Sandhofer Strasse 116 68305, Mannheim, Germany].

#### POMS questionnaire

A 36-item self-report mood scale based on the Profile of Mood States (POMS) questionnaire (18) was used to determine the current mood of the participants. The participants were asked to rate, on a scale from 0 (not at all) to 4 (extremely), how strongly their mood correlated with the various emotive words, for example, “tense”, “angry” and “unhappy”. The words were assigned to three-factor (tension, anger and depression) analytically derived subscales, and the total score of each subscale was calculated for subsequent analysis.

#### Hunger scale

A 10-point numerical rating scale (NRS), from 0 (not at all) to 9 (extremely), was used to determine the hunger scores.

#### Cold pressor test

The CPT is a standard experimental pain test used to assess pain threshold and tolerance (19), validated for use in healthy volunteers and those living with chronic pain. In this study, the CPT was set up to maintain a water temperature of 4°C, and participants were instructed to submerge their right hands into the water up to their wrists, with their fingers splayed. The participants removed their right hands until the pain became too uncomfortable (no longer tolerable). This pain tolerance, defined as the duration of time in the water before withdrawal, was measured until a safety cutoff time of 330 s.

The participants completed the CPT tasks twice each time period, referred to as the attention and distraction tasks, with a 10-min break in between. For the attention task, the participants were instructed to silently observe their hands in the cold water and focus on the pain they were feeling. For the distraction task, the participants were instructed to complete a Stroop task with their left hands (19), while their right hands were submerged. For the Stroop task, created using the PsychoPy software (version 1.84.2) with 16 colour combination options, the participants were instructed to select the word's colour rather than the word itself. The participants completed the Stroop task using their left hands as quickly and accurately as possible. Instructions were explained using the same script that was used between participants. The participants had some time to familiarise themselves with the coloured keys before completing the task. They performed a 2-min Stroop task before acting as a distraction during the CPT.

Simple randomisation was used to determine the order of the attention and distraction tasks, with neither the participants nor the researcher blinded to the order. The participants were provided with a cotton towel to dry their hands and were instructed to warm their hands before the next task.

### Statistical analysis

We used Cox regression to investigate the effect of fasting on how long the CPT was tolerated, with a pain tolerance duration censored at the 330-s safety cutoff time and taking into account the repeated measures on the same participant through a shared frailty term, with both the period and the order of fasting as fixed effects (21). Similarly, the impact of the CPT distraction task compared to the attention task and men compared to women was also estimated through the Cox regression model with shared frailties. The results are presented as hazard ratios (HRs) to express how much pain was tolerated in one group compared to another and whether any effect of fasting that differed by gender or by the distracted state was formally tested by including the terms, i.e., fasting–gender interaction and fasting–distraction interaction, in the model.

The effect of fasting on blood glucose concentration level, self-rated hunger score, and attention changes measured by the Stroop task mean response time and accuracy was estimated using linear mixed-effects models, with both period and order of fasting or non-fasting as fixed effects (22). We used Spearman’s correlation to estimate the correlation between hunger scores and blood glucose concentration levels and between blood glucose concentration levels and CPT pain tolerance.

The three subscales from the POMS questionnaire, namely, tension, anger and depression, were dichotomised at the median score (tension > 5, aggression > 2 and depression > 1) for modelling due to skewed distributions that did not resolve with log transformation. The mixed-effects Poisson regression, using the mean–variance adaptive Gauss–Hermite quadrature with 10 integration points, was used to account for the repeated measures and results presented as incidence rate ratios.

All statistical analyses were conducted in Stata version 17 (22), with 95% CI presented alongside estimates and *p* < 0.05 considered statistically significant.

A total of 25 participants, with two CPT measures (distracted and attentive states), each conducted both with and without fasting, were estimated to provide 85% power to detect a 50% longer tolerance in one fasting group compared to that in the other group (HR = 1.5), equivalent to a difference of approximately 80%, at *p* < 0.05. The median duration of pain tolerance was assumed to be approximately 200 s, censored at 330 s, with a within-person correlation of 0.75 because of the high within-person correlation seen with measures of pain tolerance.

## Results

A total of 36 participants were randomly allocated to a fasting sequence, 18 (50%) to non-fasting first and 18 (%) to fasting first, in time period 1. Among them, three participants (17%) in each group chose not to undertake the intervention or attend the first appointment. Of those who underwent the intervention, 11 (73%) who were allocated to non-fasting first were retained to time period 2, and 14 (93%) who were allocated to fasting first were retained to time period 2. No further participants were lost to follow-up from time period 2. Table 1 shows the key baseline demographic characteristics of the participants not expected to change over time, both at time periods 1 (baseline) and 2 (after crossover) by allocation sequence. These characteristics were well balanced across the randomly allocated sequences.

**Table 1:** Demographic characteristics of participants receiving intervention in time periods 1 and 2, by allocation sequence.

		Treatment sequence		
		Non-fasting first	Fasting first	Total
Period 1 (baseline)		(*n = *15)	(*n = *15)	(*n = *30)
	Women (%)	8 (53%)	7 (47%)	15 (50%)
	Mean age (years) (SD)	21 (1)	22 (3)	22 (3)
Period 2 (after crossover)		(*n = *11)	(*n = *14)	(*n = *25)
	Women (%)	6 (55%)	6 (43%)	12 (48%)
	Mean age (years) (SD)	21 (1)	22 (3)	22 (3)

The participants tolerated pain less than half the time they tolerated it when not fasting. This was equivalent to 2.4 times more likely to withdraw their hands before the 330-s CPT cutoff time (HR = 2.4, 95% CI: 1.3–4.5, *p* = 0.004) (Table 2). There was no evidence that attention or distraction had any effect (HR = 0.74, 95% CI: 0.40–1.36, *p* = 0.3) and that the effect of fasting differed by the attentive or distracted states (*p* = 0.6). In addition, there was no evidence that fasting changed the Stroop task mean response time or error rate. The fasting participants had lower levels of blood glucose concentration equivalent to 0.51 mmol/L than those of the non-fasting participants (95% CI: 0.19–0.84, *p* = 0.003) and substantially higher hunger scores when self-reported on a scale from 0 to 9 (2.7 points, 95% CI: 1.2–4.2, *p* = 0.001). There was no evidence of any effect of fasting on tension, aggression and depression, as identified from the POMS subscale scores equal to or higher than the median.

**Table 2:** Effects of fasting on the cold pressor test duration of pain tolerance, taking into account the distraction state, Stroop task mean response time and error rate, blood glucose concentration level, self-rated hunger score, profile of mood state tension and aggression and depression subscales.

	Fasted	Non-fasted	Fasted vs. non-fasted		
	Median (IQR)	Median (IQR)	HR	95% CI	*p*-value
CPT pain tolerance (s)	184 (68–330)	218 (76–330)	2.4	1.3–4.5	*p* = 0.004
	Mean (SD)	Mean (SD)	Mean difference	95% CI	*p*-value
Stroop task					
Response time (s)	0.83 (0.25)	0.77 (0.17)	0.047	−0.003 to .098	*p* = 0.063
Error rate	4.2 (6.0)	3.3 (2.8)	0.76	−1.82 to 3.34	*p* = 0.5
Blood glucose concentration level (mmol/L)	5.1 (0.5)	5.6 (0.8)	−0.51	−0.84 to −0.19	*p* = 0.003
Hunger score (0–9)	5.3 (2.7)	2.5 (2.2)	2.7	1.2 to 4.2	*p* = 0.001
** **	Mean (SD)	Mean (SD)	IRR	95% CI	*p*-value
POMS					
Tension score ≥ overall median	0.5 (0.5)	0.4 (0.5)	1.44	0.62–3.32	*p* = 0.4
Aggression score ≥ overall median	0.3 (0.5)	0.4 (0.5)	0.96	0.38–2.44	*p* = 0.9
Depression score ≥ overall median	0.6 (0.5)	0.4 (0.5)	1.49	0.67–3.29	*p* = 0.3

HR, hazard ratio; IQR, interquartile range; IRR, incidence rate ratio.

Men tolerated the pain substantially longer than women (HR = 0.021, 95% CI: 0.0027–0.16, *p* < 0.001), with men tolerating the pain up to the 330-s safety cutoff time more than half the time, but there was no evidence that the effect of fasting differed by gender (*p* = 0.9).

There were strong personal traits for pain tolerance, with the intra-person correlation for the length of pain tolerance equal to 0.66 (95% CI: 0.44–0.88) in the attentive state, 0.92 (95% CI: 0.86–0.98) in the distracted state, and 0.79 (95% CI: 0.68, 0.90) across both CPT measures.

Blood glucose concentration levels had a slight negative correlation with hunger scores (Spearman's *ρ *= −0.29, 95% CI: −0.53 to −0.01), but there was no evidence that blood glucose concentration levels were correlated with CPT pain tolerance in the attentive state (Spearman's *ρ *= 0.22, 95% CI: −0.06 to 0.46) or in the distracted state (Spearman's *ρ *= 0.17, 95% CI: −0.11 to 0.43). There was no evidence of any effect of fasting on tension, aggression and depression, as identified from the POMS subscale scores equal to or higher than the median.

## Discussion

This study showed that CPT pain tolerance significantly decreased after acute fasting and was unaffected by attentive or distracted states. Because acute fasting reduced pain tolerance, we expected the opposite action of food intake to improve pain tolerance. This was explored by Anjana and Reetu in a study involving 30 healthy volunteers which showed that CPT threshold and tolerance significantly increased half an hour after eating (23). They hypothesised the effect of the release of endogenous opioid peptides after food intake. Their findings might help explain the high prevalence of obesity in chronic pain patients as overeating behaviours act as a coping mechanism in these individuals (24–27).

Our results were only comparable to the other studies on fasting and pain in the current literature by Pollatos et al. (16). They found a significant decrease in pain threshold and tolerance measured using the pressure pain threshold (PPT) algometer test in an experimental group of 22 fasted participants compared to a control group of 12 non-fasted participants. They used a case–control design involving only women and observed sympathovagal imbalance after a 24-h fasting period (increased sympathetic activity and parasympathetic inhibition). They postulated that the autonomic imbalance and the negative mood scores in the fasting state were responsible for reducing pain tolerance. In our study, we wanted to test the response to a shorter fasting period as this is a common strategy by chronic pain individuals in their daily routines to reduce weight (28). Our crossover design with repeated within-subject measurements including both men and women made the study more robust and reduced the bias of individual variation in pain thresholds.

There was no evidence of an effect on mood within our study, demonstrating that these more acute changes in pain tolerance were independent of mood. Whilst it is known that food intake can induce a positive mood (26), it has also been shown that long-term caloric restriction can improve mood in people suffering from depression (29) and chronic pain (30), and it is highlighted that the relationship between food and mood is not simply linear. Our results with 12-h fasting showed no change in mood. In contrast, Pollatos et al. observed an increase in negative mood after 24-h fasting, which they believed could have contributed to the reduced pain tolerance. Our study highlighted that the change in pain tolerance was independent of mood. In addition, there was evidence of a beneficial effect of intermittent fasting on cognitive function (31).

There was no evidence in our study that fasting changed the Stroop task reaction time or error rate. This suggests that a 12-h fasting does not affect the focused attention of the participants. This, combined with the lack of significance of the influence of distraction on pain tolerance, suggests that the effects of fasting are not mediated by attention. Several previous studies investigating the relationship between fasting and attention showed variable findings. Some reported fasting-induced cognitive impairment (10–12), whilst others concluded no effect (13, 14) These differences were partially attributed to the differences in methodologies, concerning both fasting durations, conditions and measures of attention.

We can only speculate on the mechanisms behind fasting reducing pain tolerance at this stage. DA and 5-HT have a potential relationship between pain tolerance and food. Acute caloric restriction is known to reduce DA and 5-HT (32–34), which are both central to the endogenous analgesic descending pain pathway (35, 36). Therefore, similar to the hypothesis of Anjana and Reetu, we propose that the reduced tolerance following fasting may be due to the dysregulation of the neurotransmitters and an impaired endogenous descending pain pathway. However, these neurotransmitters increase with chronic intermittent fasting and improve cognitive function (37). Further research will help establish these complex interactions between fasting and pain in acute and chronic phases.

In addition, we wanted to investigate whether there were gender differences in pain tolerance related to fasting. Our results provide evidence that even though women have lower pain tolerance than men, there is no evidence of any different impact of fasting on pain tolerance—fasting reduced pain tolerance in both men and women. These results support the theory that women have a lower pain tolerance than men (38, 39) and provide evidence that lower pain tolerances may contribute to an increased susceptibility to developing chronic pain in women. The reasons for the differences in pain tolerances between sexes are multifactorial, encompassing physiological differences and differences in psychological processes, hormonal responses and endogenous opioid systems (38–40). Competition is another, possibly unavoidable, factor in a crossover study involving a pain task and may have contributed to gender differences in pain tolerance. Knowledge of the cutoff time for the CPT may have resulted in some participants, particularly men, becoming competitive (38). Those who reached the cutoff time in their first session may have challenged themselves to achieve it again in their second session. This will help explain why a more significant proportion of men reached the cutoff time in both the attentive and distracted sessions and fasted and non-fasted conditions compared to women.

Our study has several limitations. Firstly, the small sample size (*n* = 25 healthy volunteers) with a crossover trial design, even though it was statistically powered, would not allow for generalisation of findings, particularly applicability to individuals with chronic pain. The crossover design depends on the adequacy of the brief washout period to remove the carryover effects, but we consider this to be sufficient in this study for the outcomes used. The use of a fasting duration of 12 h may not be truly representative of intermittent fasting strategies advocated to help individuals lose weight (41, 42) and also improve pain and cognition (31, 43). A fasting period of more than 12 h is needed to enforce a metabolic switch from glycogen stores to fat mobilisation and improve cognitive function. However, compared to the 24-h duration used by Pollatos et al., we observed significant changes in capillary blood glucose levels and hunger scores sufficient to evoke fasting-induced psychological and physiological changes (16). Also, acute fasting can produce specific effects on cold sensitivity rather than pain in general. Finally, we did not investigate the effect of the type of habitual food and lifestyle on the experiment results as this was beyond the scope of our study. This can be clarified in future studies by investigating multiple stimulus modalities.

Finally, there are also limitations within the CPT methodology, and for future research, we recommend the following improvements. First, many participants reached the cutoff time of the protocol. One action to overcome this would be to recruit participants with low pain tolerances. However, excluding participants with high pain tolerance would not represent the population and may impact the results. Alternatively, we could extend the cutoff time within safe guidelines. Also, it would have been prudent to include pain visual analogue scale ratings to overcome the ceiling effect. Second, we did not standardise skin temperature. Using a skin thermistor to record skin temperature between the CPT tasks would ensure that the baseline temperatures of the participants’ hands were consistent before CPT. Circulating water, rather than non-circulating water, would help maintain a constant temperature within the ice bucket.

Further research is recommended to understand the implications of fasting on pain in chronic pain patients, particularly individuals with a BMI  > 30 kg/m^2^. Elucidating the mechanisms of the interactions between fasting, pain, and mood could help us understand the association between chronic pain conditions and eating habits. This could have implications for dieting methods in chronic pain patients and pain management in general. Chronic pain and obesity are two commonly prevalent conditions with huge healthcare costs, and effective management is likely to save costs in the system.

In summary, our study in healthy volunteers demonstrates that acute fasting significantly reduces pain tolerance and that this effect is independent of changes in attention or mood. Our results also support the theory that women have a lower pain tolerance than men. Future physiological studies in individuals with chronic pain will help us understand the relationship between fasting and pain in these individuals and help us develop better management strategies for optimising body weight and pain symptoms. We also need to understand the differential effects of acute and intermittent fasting as they do not necessarily have the same effect on pain perception and tolerance.

## Data Availability

The raw data supporting the conclusions of this article will be made available by the authors, without undue reservation.
